# Identification of compounds that suppress *Toxoplasma gondii* tachyzoites and bradyzoites

**DOI:** 10.1371/journal.pone.0178203

**Published:** 2017-06-13

**Authors:** Yuho Murata, Tatsuki Sugi, Louis M. Weiss, Kentaro Kato

**Affiliations:** 1 National Research Center for Protozoan Diseases, Obihiro University of Agriculture and Veterinary Medicine, Inada-cho, Obihiro, Hokkaido, Japan; 2 Department of Pathology, Albert Einstein College of Medicine, Bronx, New York, United States of America; University of Wisconsin Medical School, UNITED STATES

## Abstract

Drug treatment for toxoplasmosis is problematic, because current drugs cannot eradicate latent infection with *Toxoplasma gondii* and can cause bone marrow toxicity. Because latent infection remains after treatment, relapse of infection is a problem in both infections in immunocompromised patients and in congenitally infected patients. To identify lead compounds for novel drugs against *Toxoplasma gondii*, we screened a chemical compound library for anti-*Toxoplasma* activity, host cell cytotoxicity, and effect on bradyzoites. Of 878 compounds screened, 83 demonstrated >90% parasite growth inhibition. After excluding compounds that affected host cell viability, we further characterized two compounds, tanshinone IIA and hydroxyzine, which had IC_50_ values for parasite growth of 2.5 μM and 1.0 μM, respectively, and had no effect on host cell viability at 25 μM. Both tanshinone IIA and hydroxyzine inhibited parasite replication after invasion and both reduced the number of *in vitro-*induced bradyzoites, whereas, pyrimethamine, the current therapy, had no effect on bradyzoites. Both tanshinone IIA and hydroxyzine are potent lead compounds for further medicinal chemistry. The method presented for evaluating compounds for bradyzoite efficacy represents a new approach to the development of anti-*Toxoplasma* drugs to eliminate latency and treat acute infection.

## Introduction

Toxoplasmosis is caused by the pathogenic protozoan *Toxoplasma gondii*. The infection occurs in humans and other animals following ingestion of meat from infected animals (intermediate hosts) that contains tissue cysts (bradyzoites) or ingestion of food or water contaminated with oocysts shed from cats (definitive host). *T*. *gondii* disseminates as tachyzoites causing acute disease and then converts to bradyzoites that reside in tissue cysts causing a long-lived latent infection. Depending on the country and dietary habits of its population, seropositivity ranges from 6% to 77% [1]. Overall, it is estimated that a third of the world’s population is seropositive for *T*. *gondii* and has latent infection. When chronically infected patients become immunocompromised, bradyzoites can reactivate becoming tachyzoites leading to encephalitis and pneumonia [2]. Pyrimethamine and sulfadiazine, the current standard therapy for toxoplasmosis, can suppress tachyzoite growth (the acute life cycle stage) but have no effect on bradyzoites [3]. There is currently no effective treatment to eliminate *T*. *gondii* bradyzoites [4]. To identify potential drug leads to eradicate latency as well as treat the acute infection, we believe that the first step is to identify compounds that do not induce bradyzoite differentiation and are effective against bradyzoites.

Screening an unbiased compound library is a powerful tool for the identification of effective compounds against pathogens without knowing in advance the actual target proteins. Such drug-repurposing strategies involving other protozoan parasites has also successfully identified effective compounds [5]. Furthermore, the predicted mode of action of the various compounds in a validated chemical compound library facilitates an improved understanding of new anti-parasitic compounds when effective compounds are identified during the screening process.

Screening for effective compounds that do not induce bradyzoites requires the screening method including an evaluation of bradyzoite differentiation. “Compound 1”, which was firstly identified as a coccidian cGMP dependent protein kinase inhibitor [6], effectively suppressed the parasitic infection in *Toxoplasma gondii* acute model [7], later it was identified to induce bradyzoite differentiation [8], further suggesting the requirement of evaluation of bradyzoite differentiation. Several reporter parasites have been previously described that can be used to evaluate bradyzoite differentiation, including those that utilize fluorescent proteins [9], β-galactosidase enzyme activity [10], or luciferase activity [11, 12]. In the screening method described here, we utilized PLK/DLUC_1C9 [12] to evaluate parasite growth as ascertained by the amount of Renilla luciferase activity expressed under the control of the tubulin promoter and to evaluate bradyzoite differentiation as determined by the amount of firefly luciferase activity expressed under the bradyzoite-specific BAG1 promoter [12]. A validated chemical library was screened for anti-*Toxoplasma* activity and host cell cytotoxicity. Compounds with good anti-*Toxoplasma* activity and low host cell toxicity were then further evaluated for their effects on bradyzoite growth and differentiation. This screening led to the identification of tanshinone IIA and hydroxyzine as novel anti-*Toxoplasma* compounds that were active against both tachyzoites and bradyzoites.

## Materials and methods

### Compounds

A validated chemical compound library (Prestwick and LOPAC chemical library) was provided by the Drug Discovery Initiative (The University of Tokyo, Tokyo, Japan; http://www.ddi.u-tokyo.ac.jp/en/). Pyrimethamine, fluphenazine, and perospirone (Wako, Osaka, Japan); perphenazine, mefloquine, tanshinone IIA, and butein (Tokyo Chemical Industry, Tokyo, Japan); hydroxyzine and penitrem A (LKT Labs, MN, USA; (±)-terfenadine and AM404 (R&D Systems, MN, USA); domperidone, PQ-401, bromocriptine, and omeprazole (Sigma-Aldrich, MO, USA); niguldipine (Focus Biomolecules, PA, USA); MC-1293 (Santa Cruz Biotechnology, TX, USA); and entinostat (ChemScene Chemicals, NJ, USA) were used for secondary screening as described below.

### Toxoplasma gondii in vitro culture

Vero cells (RIKEN BioResource Center: RCB0001) or human foreskin fibroblasts (HFF) (ATCC: SCRC-1041) were used as host cells for *T*. *gondii* culture. Vero cells were maintained in DMEM (Nissui Pharmaceutical, Tokyo, Japan) supplemented with 5% FBS, L-glutamine, penicillin, and streptomycin. HFF cells were maintained in DMEM supplemented with 10% FBS, L-glutamine, penicillin, and streptomycin. RH-2F (ATCC: 50839), PLK/DLUC_1C9 [12], and Pru*Δku80Δhxgprt* [13] were maintained as previously described [12].

### Toxoplasma gondii growth assay

In the primary screening, purified PLK/DLULC_1C9 tachyzoites were incubated with Vero cells at a multiplicity of infection (MOI) of 0.5. After a 2-h incubation, the infected host cells were trypsinized to obtain a cell suspension and then these suspended infected cells were inoculated into 96-well optical bottom plates containing the compound library. Each well was inoculated with 2500 cells in 25 μL of culture medium. Each well had been previously inoculated with 125 nL of DMSO containing compound at a concentration of 2 mM. Following cell inoculation, each well therefore contained 0.5% DMSO and 10 μM compound. After 48 h of culture, Renilla luciferase activity was measured by using Renilla-Glo (Promega, WI, USA) following the manufacturer’s instructions. Each plate had eight DMSO (negative controls) and eight 10 μM pyrimethamine (positive control) wells. The Z’-value [14] for each plate was calculated from the Renilla luciferase activity in the negative and positive control wells. Compounds that showed >90% parasite growth inhibition in the plates with a Z’-value of >0.5 were selected for confirmation and secondary screening.

In the confirmatory screening, we utilize an RH strain β-galactosidase reporter parasite termed RH-2F (a type I strain) [15]. Briefly, RH-2F parasites were purified from freshly lysed infected host cells and used to infect confluent HFF cells cultured in 96-well plates. Five hundred parasites were added to each well, along with each test compound and 50 μL of culture medium. Plates were then incubated for 10 min at room temperature followed by an incubation at 37°C for 48 h in a CO_2_ incubator.

To measure the effects of tanshinone IIA and hydroxyzine on intracellular parasite growth, parasites were allowed to invade for 2 h, uninvaded parasites were washed away, and medium with compounds was added. After 48 h, the plates were frozen at -80°C. Plates were then thawed just prior to the measurement of beta-galactosidase activity. Beta-Glo (Promega) was used to measure β-galactosidase activity following the manufacturer’s instructions.

### Host cell cytotoxicity assay

Host cells were treated with compounds at the same concentration used for screening (i.e., 0.5% DMSO and 10 μM compound). Vero cells and HFF cells were both used as host cells because both cell types were used for the parasite screening. After incubation for 48 h in DMEM supplemented with 5% FBS, L-glutamine, penicillin, and streptomycin, cell viabilities were measured by using a Cell-Titer Glo kit (Promega) following the manufacturer’s instructions. Control wells were treated with DMSO alone in culture medium to calculate the 100% viability for each cell type.

To evaluate the host cell viability in bradyzoite differentiation condition, HFF cells were incubated for 48 h in DMEM containing 25 mM HEPES pH 8.1 and 1% FBS and measured the viability as described above.

### Bradyzoite differentiation assay

To measure bradyzoite induction activities of tanshinone IIA and hydroxyzine, PLK/DLUC_1C9 parasites were purified and then inoculated at a concentration of 2.0 x 10^4^ parasites/well into 12-well plates containing confluent monolayers of HFF cells. Cells were incubated for 24 h after infection, then compounds were added, and the cells were incubated for 48 h. After incubation, the cells in each well were lysed using 200 μL of Passive Lysis Buffer (Promega) and the lysates were stored at -80°C. For the luciferase assay, 25 μL of lysate was used and the Renilla and firefly luciferase activities were measured by using Dual-Glo (Promega) according to the manufacturer’s instruction.

To measure the effect of tanshinone IIA and hydroxyzine on spontaneously induced *in vitro* bradyzoites, an immunofluorescence-based assay was conducted. Pru*Δku80Δhxgprt*, which expresses GFP under the control of a bradyzoite-specific gene LDH2 promoter, [13] was used. HFF host cells were seeded to a 96-well glass bottom plate (MGB096-1-2-LG-L, Brooks Automation, MA, USA) and cultured until confluent. Parasites were freshly lysed by passing them through 27 G needles and were purified by passing them through a 5-μm-pore filter. Five thousand parasites in 100 μL of culture medium were added and mixed with an equal volume of compound-containing medium to give final drug concentrations of 10, 2, 0.4, and 0.08 μM in 0.2% DMSO. After incubation at room temperature for 10 min, the infected host cells were incubated for 48 h at 37°C in a 5% CO_2_ incubator. After incubation, the cells were fixed with 4% paraformaldehyde in PBS for 20 min, quenched, and permeabilized with 0.2 M glycine and 0.2% TritonX-100 in PBS for 20 min, and then blocked with 3% BSA and 0.1% Tween 20 in PBS (blocking buffer); each procedure was performed at room temperature and was followed by washes with PBS three times. All parasites were stained with an anti-*Toxoplasma* mouse antibody (antisera produced in ME49-infected mice), and bradyzoites were detected with an anti-GFP rabbit monoclonal antibody (Life Technologies). Secondary antibodies for mouse (Alexa633 conjugated) and rabbit (Alexa488 conjugated) and DAPI were used to develop the fluorescent signal. Stained cells on 96-well plates were analyzed with a CompuCyte iCys Research Imaging Cytometer (Beckman Coulter, IN, USA).

To measure the effect of tanshinone IIA and hydroxyzine on *in vitro-*induced bradyzoites, we used PLK/DLUC_ 1C9 parasites and the Dual-Glo luciferase assay. Briefly, HFF cells in 96-well plates were infected with 1 x 10^4^ parasites/well and incubated for 3 days with ambient CO_2_ condition in DMEM containing 25 mM HEPES pH 8.1 and 1% FBS to induce bradyzoites. Induced bradyzoites were treated with 10, 5, 2.5, and 1 μM compounds under bradyzoite-inducing conditions. After 48 h, luciferase activities were measured by using the Dual-Glo assay system (Promega) and normalized by host cell number.

## Results

### Screening of the chemical compound library for anti-Toxoplasma efficacy

**Fig 1:**
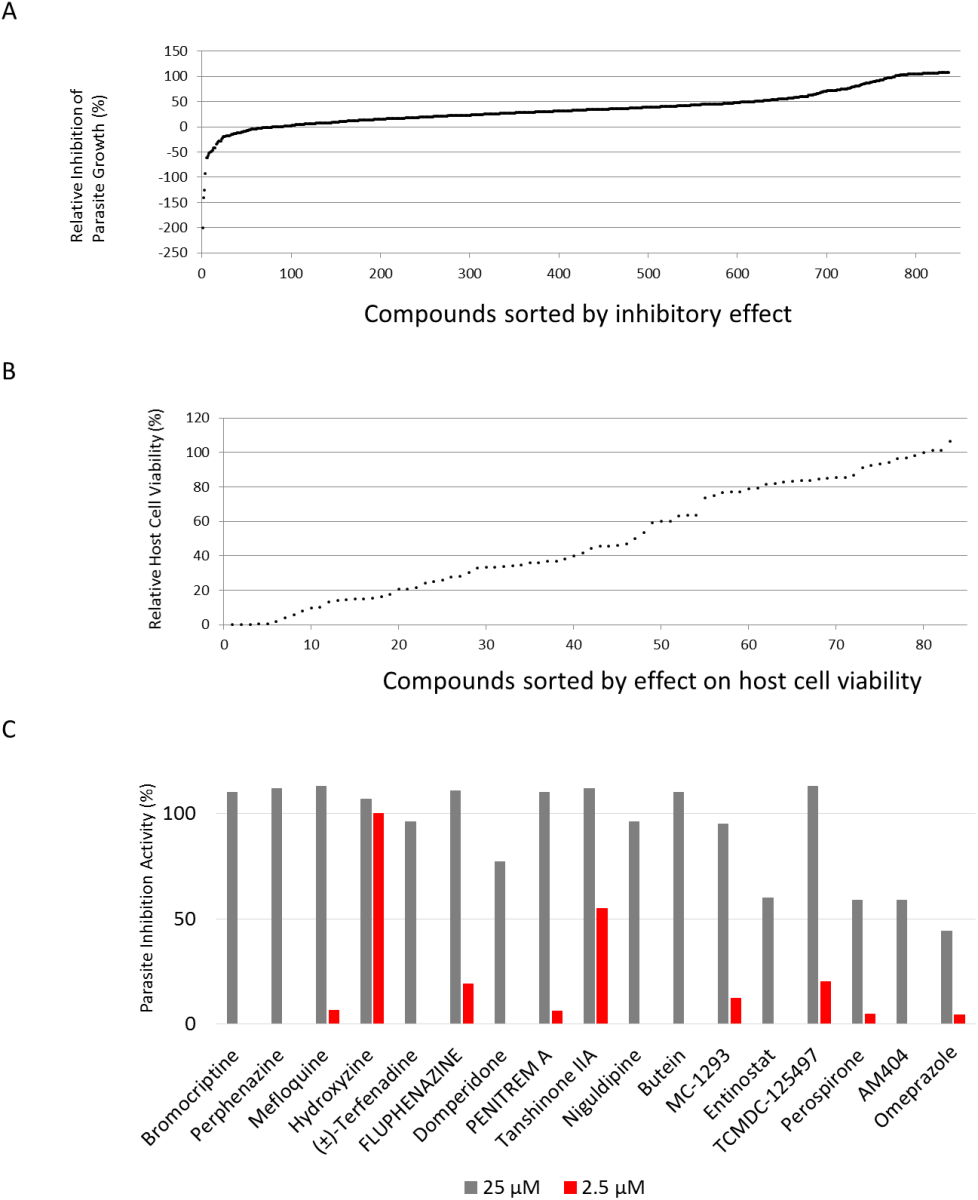
Primary screening for anti-*Toxoplasma* activity and cytotoxicity. **(A)** PLK/DLUC_1C9-infected Vero cells were incubated with 10 μM test compounds (837 compounds) for 48 h (see Materials and Methods). Renilla luciferase, expressed by PLK/DLUC_1C9 *T*. *gondii*, was measured, and inhibition was calculated with DMSO as a negative control (0% inhibition) and 10 μM pyrimethamine as a positive control (100% inhibition). Compounds in the plates that had a Z’-value ≥0.5 are presented sorted by inhibitory effect. **(B)** Compounds that showed ≥90% parasite inhibition (83 compounds) were then screened for effects on host cell viability (see Materials and Methods). Compounds were screened at a concentration of 10 μM. Cell viability values were calculated relative to background values (0% viability) and the DMSO negative control (100% viability). **(C)** Parasite growth inhibition activities were shown at 25μM (gray bars) and 2.5μM (red bars). Tanshinone IIA and hydroxyzine had >50% growth inhibitory effect on parasites.

A total of 828 compounds were screened at 10 μM for their *T*. *gondii* growth inhibitory effects. Luciferase activities of the wells added only DMSO were calculated as 0% inhibition and those of the wells added 10 μM Pyrimethamine, which were enough concentration to show maximum inhibitory effect for parasite growth (S1 Fig), were calculated as 100% growth inhibition. Of these, 83 compounds had a growth inhibition rate of >90% (Fig 1A). Host cell viability was examined for these 83 compounds (Fig 1B), and 24 of them did not significantly suppress host cell viability (80%, viability) (Table 1), suggesting that the effect of these compounds on parasite growth inhibition was not a consequence of host cell cytotoxicity and that these compounds could be considered to be leads for anti-*Toxoplasma* therapy.

**Table 1:** Hit compounds from 1st screening.

compound	Parasite Inhibition (%)(a)	Host Viability (%)(b)
Bromocriptine	105	85
Perphenazine	104	84
Mefloquine	101	87
Isoconazole	104	101
Hydroxyzine	97	101
(±)-Terfenadine	104	85
Clotrimazol	99	94
FLUPHENAZINE	103	83
Domperidone	93	94
Hycanthone	97	82
Propidium Iodide	91	91
PENITREM A	105	79
Tanshinone IIA	91	83
Niguldipine	103	77
N-Hydroxy-N2-isobutyl-N2-[(4-methoxyphenyl)sulfonyl]glycinamide	101	98
N-{[(2-Methyl-2-propanyl)oxy]carbonyl}glycyl-N-[(2S)-3-methyl-1-oxo-2-butanyl]-L-valinamide	105	106
Butein	96	93
MC-1293	103	85
Entinostat	91	83
TCMDC-125497	106	82
Perospirone	100	96
Omeprazole	92	97
AM404	103	100
(2Z)-2-(1,3-Benzodioxol-5-yl)-4-(4-methoxyphenyl)-4-oxo-3-(3,4,5-trimethoxybenzyl)-2-butenoic acid	93	85

(a) Parasite growth inhibition values from 1st screening with PLK/DLUC_1C9 with 10 μM concentration. DMSO control was calculated as 0% inhibition and 10 μM Pyrimethamine control was calculated as 100% growth inhibition.

(b) Host Cell viability values from 1st screening with Vero cells with 10 μM concentration. DMSO control was calculated as 100% cell viability.

### Validation of the screening method

**Fig 2:**
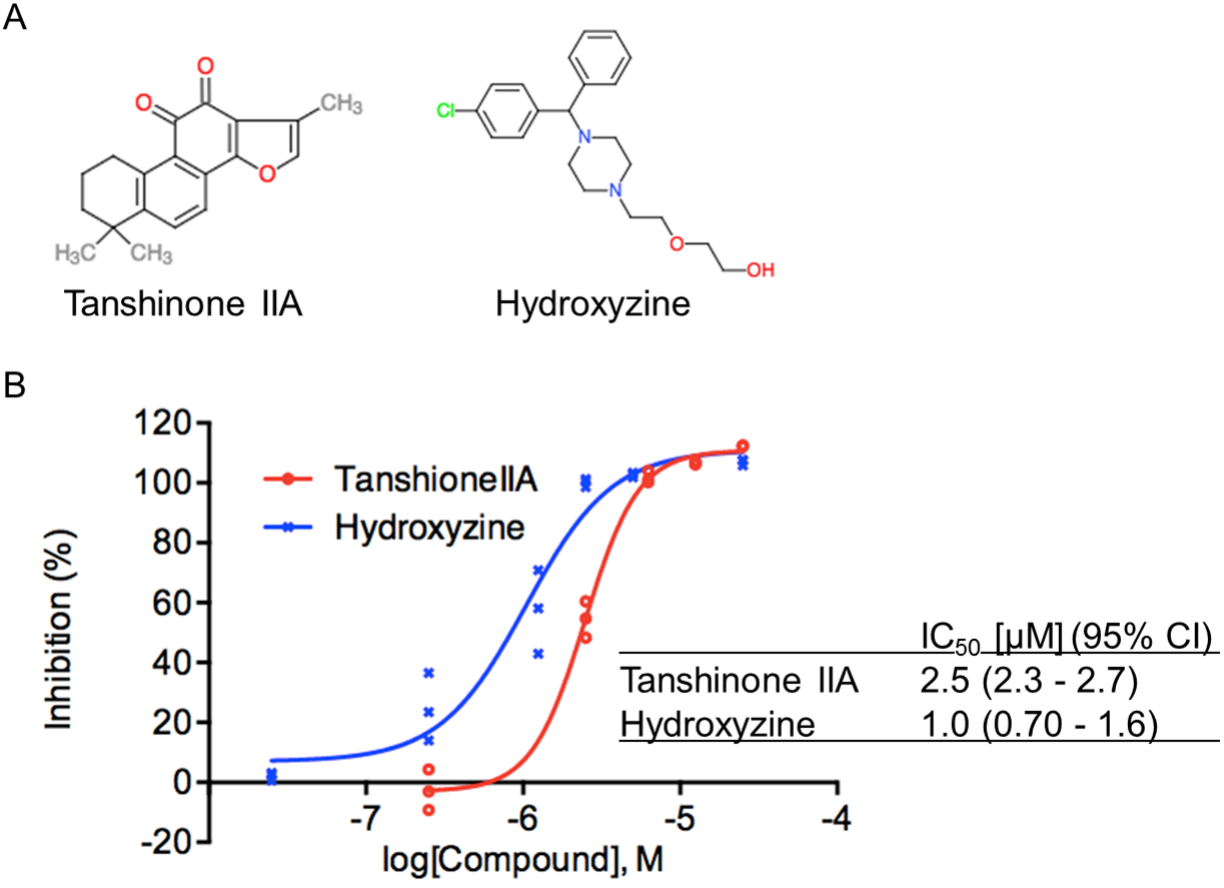
Tanshinone IIA and hydroxyzine display dose-dependent inhibitory effects on *Toxoplasma gondii* growth. **(A)** Structures of tanshinone IIA and hydroxyzine. **(B)** Dose-response response curves and IC_50_ values for tanshinone IIA and hydroxyzine. HFF cells infected with RH-2F parasites were incubated with various concentrations of the test compounds and parasite growth was measured by using the β-galactosidase assay. Inhibition rates were calculated using DMSO as a negative control (0% inhibition) and 10 μM pyrimethamine as a positive control (100% inhibition).

Previous studies revealed that antifungal agents (Isoconazole and Clotrimazole) are effective to inhibit the growth of *T*. *gondii* [16, 17]. Propidium iodide is not suitable for treatment of toxoplasmosis because this agent cannot pass through the cell membrane. Therefore, we confirmed that the novel candidates were potent agents for treatment of toxoplasmosis. Among the available hit compounds, 17 including mefloquine, dopamine and histamine receptor antagonists, HDAC inhibitors, and a COX1 potential inhibitor (AM404), were evaluated for their ability to suppress the parasite growth using the different host and parasite strains from the strain for first screening, a type I strain parasite (RH-2F) in HFF host cells (Table 2 and Fig 1C). All of the test compounds demonstrated at least 50% inhibition at 25 μM in this validation assay (Table 2), suggesting that the effect of these compounds was not dependent on the specific parasite genotype and was not host-cell specific. Two compounds, tanshinone IIA and hydroxyzine (Fig 2A), were particularly potent, demonstrating over 50% inhibition at 2.5 μM, compared with 10 μM pyrimethamine treatment, and almost no effect on host cell viability at 25 μM (100.5% and 74.8% host cell viability, respectively, compared with the DMSO solvent control, Table 2). For these two compounds dose-dependent parasite growth inhibition curves were obtained (Fig 2B). Hydroxyzine had an IC_50_ value of 1.0 μM (0.70 μM– 1.60 μM; 95% Confidence Interval). Tanshinone IIA had an IC_50_ value of 2.5 μM (2.3 μM– 2.7 μM; 95% Confidence Interval) (Fig 2B). These data suggest that tanshinone IIA and hydroxyzine are most potent drug candidates among hit compounds.

**Table 2:** Effects of hit compounds on RH-2F tachyzoites and HFF cells.

	Parasite inhibition (%) (a)		Host cell viability (%) (b)	
Compound	25 μM	2.5 μM	25 μM	2.5 μM
Bromocriptine	110	-8.0	93	98
Perphenazine	112	-6.8	65	95
Mefloquine	113	6.5	48	94
Hydroxyzine	107	100	100	102
(±)-Terfenadine	96	-12	0.1	96
FLUPHENAZINE	111	19	84	104
Domperidone	77	-7.2	83	94
PENITREM A	110	6.0	34	102
Tanshinone IIA	112	55	75	99
Niguldipine	96	-19	84	99
Butein	110	-11	91	102
MC-1293	95	12	95	98
Entinostat	60	-20	91	92
TCMDC-125497	113	20	78	99
Perospirone	59	4.5	101	102
AM404	59	-11	86	93
Omeprazole	44	4.3	100	100

(a) Parasite inhibition of test compounds compared with that of 10 μM pyrimethamine was examined by infecting HFF host cells with RH-2F and treating the cells with the test compounds for 48 h.

(b) HFF host cell viability was assessed after 48 h of compound treatment.

### Tanshinone IIA and hydroxyzine inhibit intracellular parasite growth

**Fig 3:**
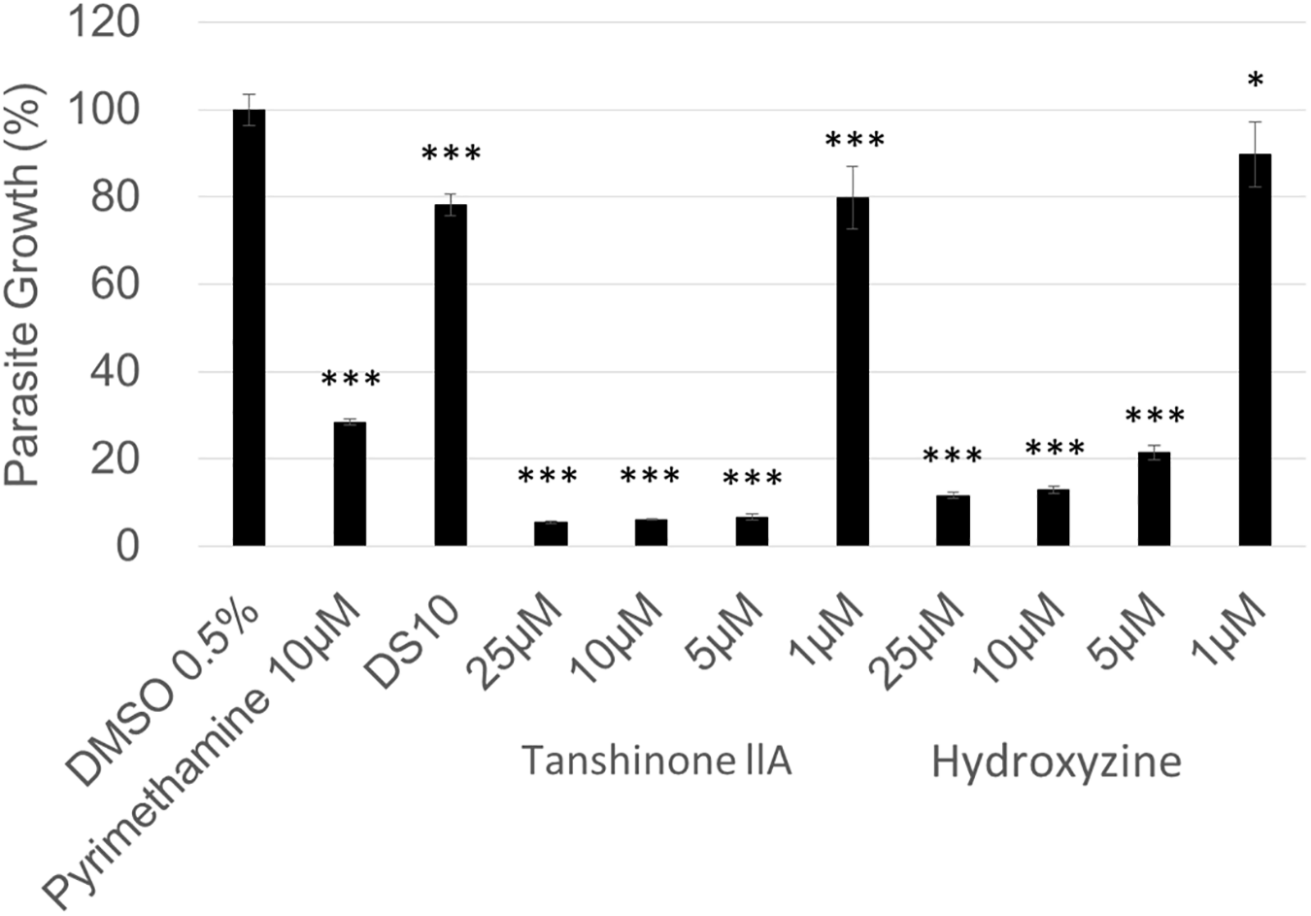
Evaluation of the inhibitory effects of tanshinone IIA and hydroxyzine on parasite replication after invasion. 1.0 μM hydroxyzine, 2.5 μM tanshinone IIA, 10 μM pyrimethamine, 25 mg/ml Dextran Sulfate 10 kDa fraction (DS10), or DMSO solvent control was added to parasites after washing away uninvaded parasites at 2 h post-infection. Infected host cells were incubated for 48 h and β-galactosidase activity was measured to estimate the parasite number. The DMSO control without parasite washing was set as 100%. Means ± SD from triplicate experiments are shown. The statistical difference between the DMSO control and each compound was evaluated by using Dunnett’s test. *p<0.05, *** *p*< 0.001.

To clarify whether these compounds are active against intracellular parasites, we added them to parasite cultures after host cell invasion. When the compounds were added after the 2 h invasion window, tanshinone IIA and hydroxyzine reduced parasite growth in a dose dependent manner similar to the positive control pyrimethamine (which is well known to inhibit purine *de novo* synthesis and intracellular growth); however, Dextran Sulfate 10 kDa, which is known to inhibit parasite invasion [18], did not affect growth (Fig 3). Parasites that had not invaded were removed by washing following the 2 h invasion period in this assay, indicating that the reduced beta-galactosidase signals came from the parasite wells that were washed (Fig 3).

### Effects of tanshinone IIA and hydroxyzine on bradyzoites

**Fig 4:**
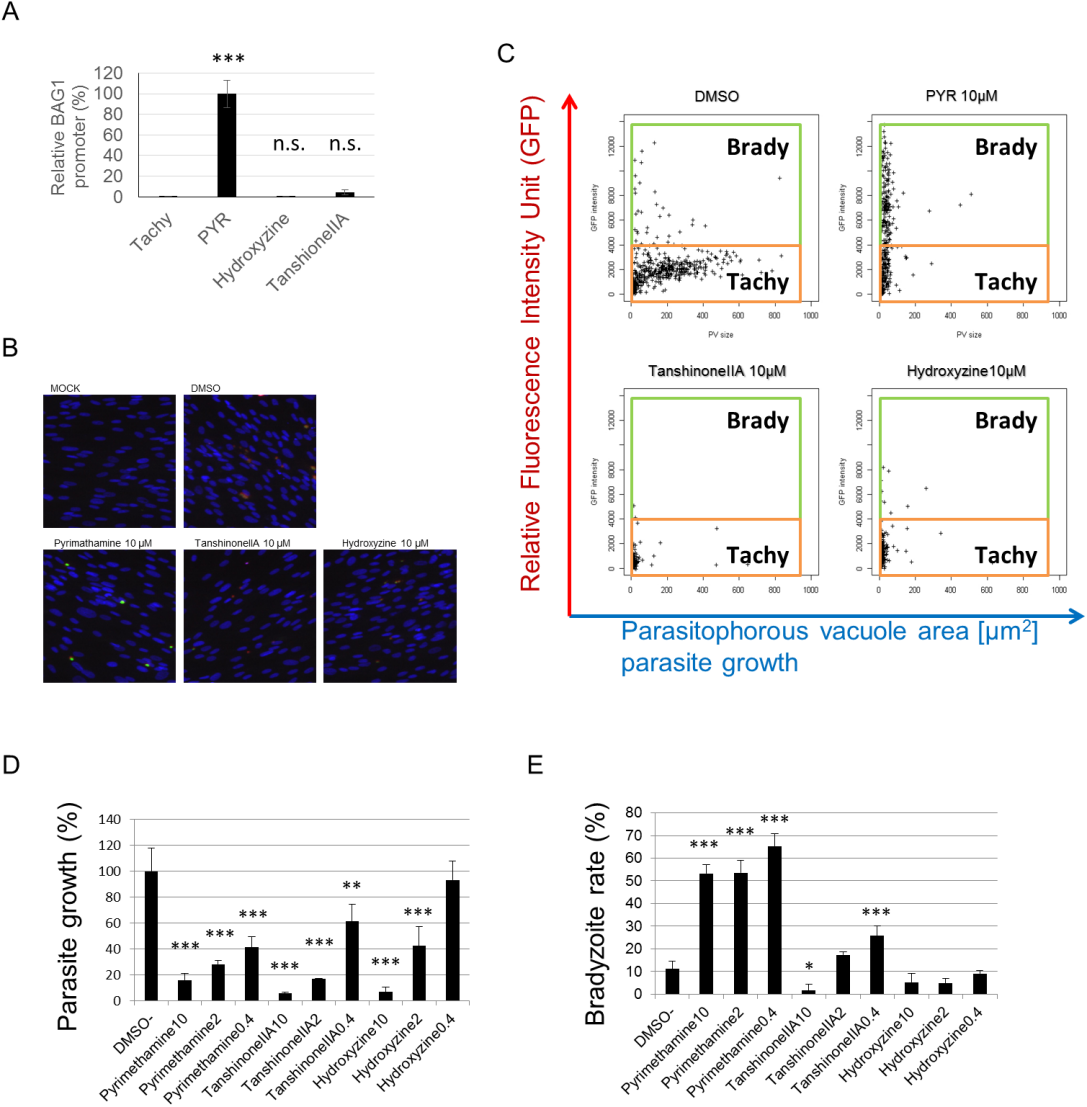
Bradyzoite-inducting effects of tanshinone IIA and hydroxyzine compared with those of pyrimethamine. (A) PLK_DLUC_1C9 parasites were inoculated onto a monolayer of HFF cells and incubated for 24 h before test compounds (5 μM pyrimethamine, 1 μM hydroxyzine, 2.5 μM tanshinone IIA, or DMSO solvent control) were added. Infected cells were treated with test compounds for 48 h and then relative BAG1 promoter activity was calculated by dividing the Firefly luciferase activity, expressed by the BAG1 promoter, by the Renilla luciferase activity, expressed by the TUB promoter. Relative BAG1 promoter activity was compared with that of pyrimethamine-treated samples as shown. Means ± SD are from triplicate experiments. The statistical difference between the DMSO solvent control group (tachyzoite) and each compound was evaluated by using Dunnett’s test, *** *p*< 0.001. (B–E) To evaluate the effects of the test compounds on spontaneously differentiated bradyzoites, Pru*Δku80Δhxgprt* were inoculated into host HFF cells with the compounds. At 48 h post infection, the infected host cells were fixed and stained with an anti-*Toxoplasma* mouse antibody and an anti-GFP antibody and 25 images were taken for each well by automated fluorescence microscopy. Representative images of the DMSO-, 10 μM pyrimethamine-, tanshinone IIA-, and hydroxyzine-treated wells are shown in (B). Each parasitophorous vacuole (PV), which was detected by the anti-Toxoplasma antibody, was quantified by size and GFP intensity. (C) Dot plots of the detected PV profiles for the DMSO, 10 μM pyrimethamine, tanshinone IIA, and hydroxyzine groups. To determine for the rate of bradyzoite induction, a threshold value of 4000 for the relative fluorescent units was set, as indicated in (C). Means ± SD from three wells for each group were calculated for the total PV size in the 25 images (parasite growth) (D) and GFP-positive PV rate (E). The statistical difference between the DMSO control and each compound was evaluated by using Dunnett’s test. * *p*<0.05, ** *p* < 0.01 and *** *p* <0.001.

To confirm whether the compounds shows preferential effects on each parasite stage, tachyzoite or bradyzoite, we assessed the ratio of bradyzoites when the parasites were incubated with the IC_50_ concentrations of tanshinone IIA and hydroxyzine. The relative BAG1 promoter activity (which reflects the bradyzoite ratio of treated parasites) compared with 5 μM pyrimethamine treatment was 0.3% (DMSO control), 0.4% (1 μM hydroxyzine), and 4.7% (2.5 μM tanshinone IIA) (Fig 4A). To validate these effects on the bradyzoite, we used another parasite strain, Pru*Δku80Δhxgprt*, which is known to undergo spontaneous bradyzoite differentiation. After 48 h of treating the infected host cells with various concentrations of test compounds, parasites were stained for the total parasite and the bradyzoite reporter gene GFP (see Fig 4B and S2 Fig for representative images). Images were taken with a computer-assisted fluorescent microscopy and each parasite vacuole in the images was analyzed for size and GFP intensity (Fig 4C). In the DMSO treatment well, there were two populations of GFP-positive and GFP-negative parasites (Fig 4C upper left) and in the pyrimethamine treatment well, the GFP-positive population increased (Fig 4C upper right). Parasite growth was estimated by the total parasite vacuole size in the images, and bradyzoite differentiation was estimated by the rate of bradyzoites showing a GFP signal over the threshold value, as shown in Fig 4C. All of the test compounds, pyrimethamine, tanshinone IIA, and hydroxyzine, reduced parasite growth in a concentration-dependent manner (Fig 4D). Pyrimethamine induced bradyzoite differentiation, whereas tanshinone IIA and hydroxyzine reduced parasite growth and did not induce bradyzoite differentiation even at high concentrations, compared with the DMSO treatment wells (Fig 4E). These data demonstrate that tanshinone IIA and hydroxyzine are not selective for the tachyzoite stages and have no bradyzoite induction effects.

**Fig 5:**
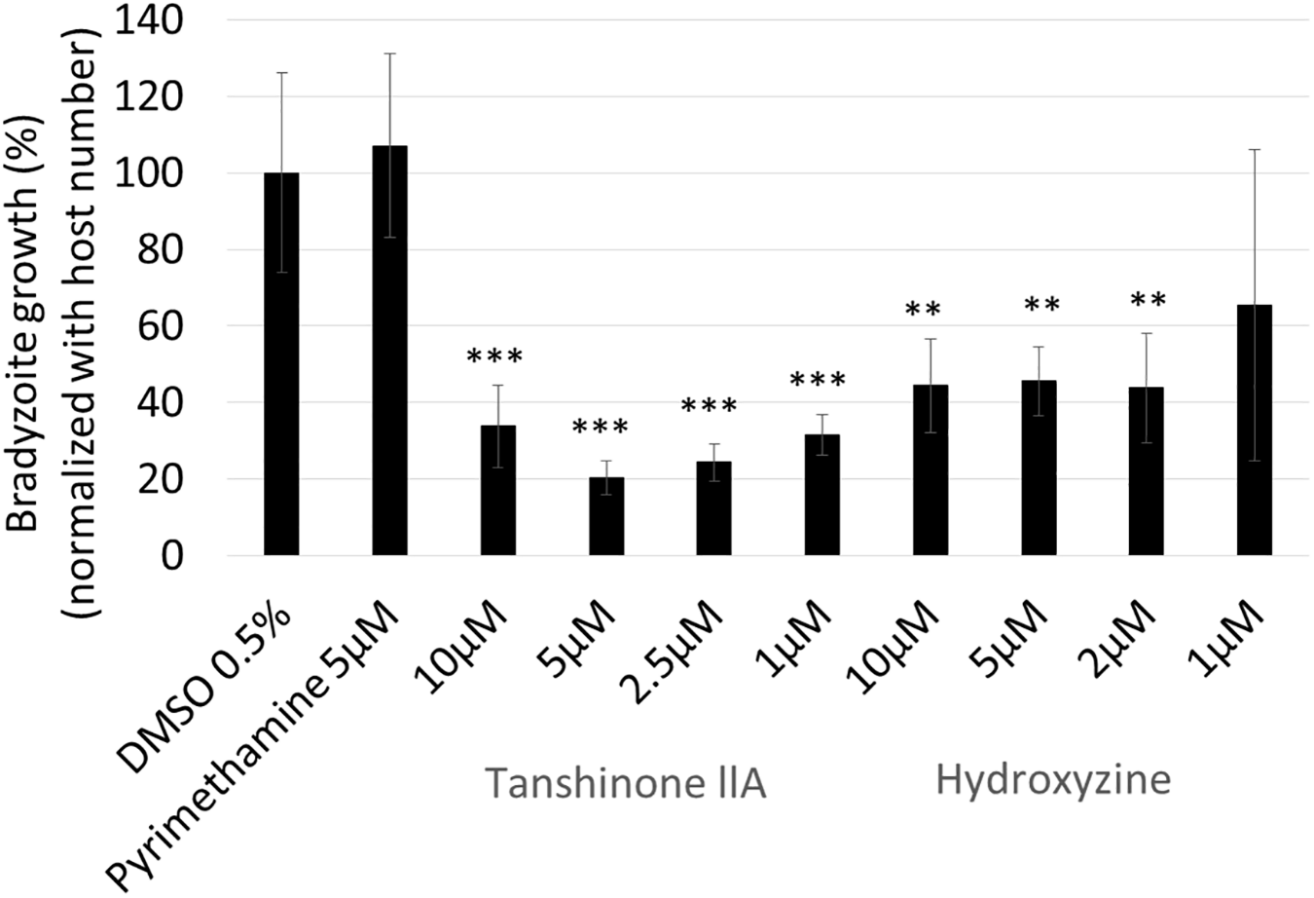
Tanshinone IIA and hydroxyzine reduced the number of *in vitro-*differentiated bradyzoites. PLK_DLUC_1C9 *T*. *gondii* were inoculated onto a monolayer of HFF cells and incubated for 2 h before undergoing bradyzoite induction for 3 days. After bradyzoite induction, compounds, as indicated, were added to the medium and infected host cells were incubated for 2 days under bradyzoite culture conditions. Firefly luciferase activity, under the control of the bradyzoite-specific BAG1 promoter, was measured and normalized to non-treated control (DMSO) wells. Bradyzoite growth rate was normalized with host cell number. Means ± SD from triplicate wells are shown. The statistical difference between the DMSO control and each compound was evaluated by using Dunnett’s test. ** *p* < 0.01 and *** *p* <0.001.

To determine whether tanshinone IIA and hydroxyzine could affect the *in vitro-*differentiated bradyzoites, we treated the intermediately differentiated bradyzoites (cultured in bradyzoite inducing condition for 3 days) with these compounds and measured the bradyzoite number. As the pH stress induced the bradyzoite differentiation by having an effect on host cells [2], we first tested the host cell viability under the bradyzoite-inducing condition (S3A Fig). Tanshinone IIA slightly reduced the host cell number, but hydroxyzine did not affect the host cell viability. Next, we evaluated firefly luciferase activity under the control of the bradyzoite-specific BAG1 promoter as a measure of bradyzoite number (S3B Fig), and normalized by host cell number (Fig 5). Both tanshinone IIA and hydroxyzine reduced the bradyzoite number relative to treatment with DMSO or pyrimethamine. In particular, tanshinone IIA reduced the bradyzoite number most effectively at the lowest dose (1 μM). These data suggest that tanshinone IIA and hydroxyzine can inhibit the growth of intermediately differentiated bradyzoites, whereas the current recommended drug pyrimethamine cannot reduce bradyzoite numbers.

## Discussion

Drug repurposing or the screening of validated compound libraries has successfully identified potential targets for the treatment of parasitic diseases (reviewed in [5]). We found that some of the compounds we identified in our library screen had similar functions. In particular, our screen identified antagonists of neurotransmitter receptors as new potential anti-*Toxoplasma* compounds, including dopamine receptor antagonists (e.g., perphenazine, fluphenazine, domperidone, and perospirone) and histamine receptor antagonists (e.g., hydroxyzine and (±)-terfenadine), which have been reported to be effective against *T*. *gondii* growth [19]. Our study also showed that compounds that may inhibit the growth of cancer cells (e.g., tanshinone IIA [20], butein [21], and entinostat [22]) could also reduce the parasite number. Although we have not yet assessed the mechanism by which these anti-*Toxoplasma* compounds block parasite growth, the mechanism may reveal a new target for the control of *Toxoplasma gondii*.

While therapy exists for the treatment of toxoplasmosis, it is suboptimal and there is a need for new therapeutic agents with fewer side effects and for agents that are active against latent infection. The use of a bradyzoite reporter parasite facilitates the evaluation of compounds for their effects on both tachyzoites and bradyzoites. We identified two compounds, tanshinone IIA and hydroxyzine, that had a greater effect on bradyzoites (chronic infection) than that of pyrimethamine yet still had significant anti-tachyzoite activity (acute infection).

Hydroxyzine is an FDA-approved antihistamine that binds Histamine Receptor 1 [23]. It is widely used for the treatment of pruritus and anxiety, and for sedation. Our screen revealed that antagonists of dopamine and histamine receptors are effective inhibitors of *T*. *gondii* growth. On the other hand, bromocriptine, which is dopamine receptor agonist could also inhibit the growth of *T*. *gondii*. Other agonists of host cell receptors have been reported to have anti-parasite effects [24, 25]. Although these compounds have known targets on host cells, the anti-*Toxoplasma* effects of the dopamine receptor agonist pimozide cannot be reversed by the addition of dopamine [19], suggesting that these host cell receptor agonists have effects beyond the primary target pathway.

Tanshinone IIA has been reported to have a variety of functions, typified by suppression of inflammation and of cancer cell replication [26]. For example, one study revealed that tanshinone IIA can induce cell cycle arrest and apoptosis in lung cancer cells [20]. In our study, tanshinone IIA effectively reduced the number of bradyzoites, which replicate very slowly, suggesting that the mechanism against *Toxoplasma* might be the induction of cell death, not cell cycle arrest. Tanshinone IIA has been reported to have anti-*Plasmodium* effects [27], suggesting that there may be a common target pathway among the Apicomplexa.

The method described in this paper represents a screening strategy to identify anti-*Toxoplasma* compounds that have both tachyzoite and bradyzoite efficacy and could be used to treat both acute and latent infection. The *T*. *gondii* parasites that reside in *in vivo* brain cysts and cause latent infection have been demonstrated to be dynamically replicating entities [28]. Several drugs that target these replicating latent parasites have recently been identified and include a bumped kinase inhibitor [29], which targets *T*. *gondii* CDPK1, and endochin-like quinolones [30], which inhibit the cytochrome bc1 complex. The activity of these compounds toward bradyzoites suggests that therapeutic agents can be found that will treat latent infection with this parasite. Additional screens using the methods we present here should help identify additional leads, and it is likely that one of these leads will result in a clinically effective new therapy that will treat both acute and chronic infection.

## Conclusions

We identified tanshinone IIA and hydroxyzine as effective inhibitors of the growth of tachyzoites. While the current recommended drug pyrimethamine induces bradyzoite differentiation, tanshinone IIA and hydroxyzine did not increase the bradyzoite population. Moreover, these compounds could inhibit the growth of intermediately differentiated bradyzoites. Our data indicate that tanshinone IIA and hydroxyzine represent novel lead compounds to treat acute toxoplasmosis as well as prevent reactivation of the latent infection.

## Supporting information

S1 Fig Dose response curve for pyrimethamine. HFF cells infected with RH-2F parasites were incubated with various concentrations of pyrimethamine and parasite growth was measured by using the β-galactosidase assay. Inhibition rates were calculated using wells added DMSO (0% inhibition) and mock-infected wells (100% inhibition).
(TIF)

S2 Fig Parasite growth assay combined with the bradyzoite rate estimation. High resolution and individual channel of the images in Fig 3B are shown. PYR: Pyrimethamine, HXZ: Hydroxyzine, TIIA: TanshinoneIIA.
(TIF)

S3 Fig Effect of tanshinone IIA and hydroxyzine on host cells and parasites. **(A)** HFF cells were incubated with bradyzoite condition (pH8.1, 0.03% CO_2_) with tested compounds. After 48 hours, host cell viabilities were measured. **(B)** PLK_DLUC_1C9 *T*. *gondii* were inoculated onto a monolayer of HFF cells and incubated for 2 h before undergoing bradyzoite induction for 3 days. After bradyzoite induction, compounds, as indicated, were added to the medium and infected host cells were incubated for 2 days under bradyzoite culture conditions. Firefly luciferase activities, under the control of the bradyzoite-specific BAG1 promoter, were measured and normalized to non-treated control (DMSO) wells. The statistical difference between the DMSO control and each compound was evaluated by using Dunnett’s test. ** *p* < 0.01 and *** *p* <0.001.
(TIF)
