# Supplementary figures and images for: Identification of compounds that suppress Toxoplasma gondii tachyzoites and bradyzoites
Source: PLoS One. 2017 Jun 13;12(6):e0178203. doi: 10.1371/journal.pone.0178203 (PMC5469451; doi:10.1371/journal.pone.0178203)

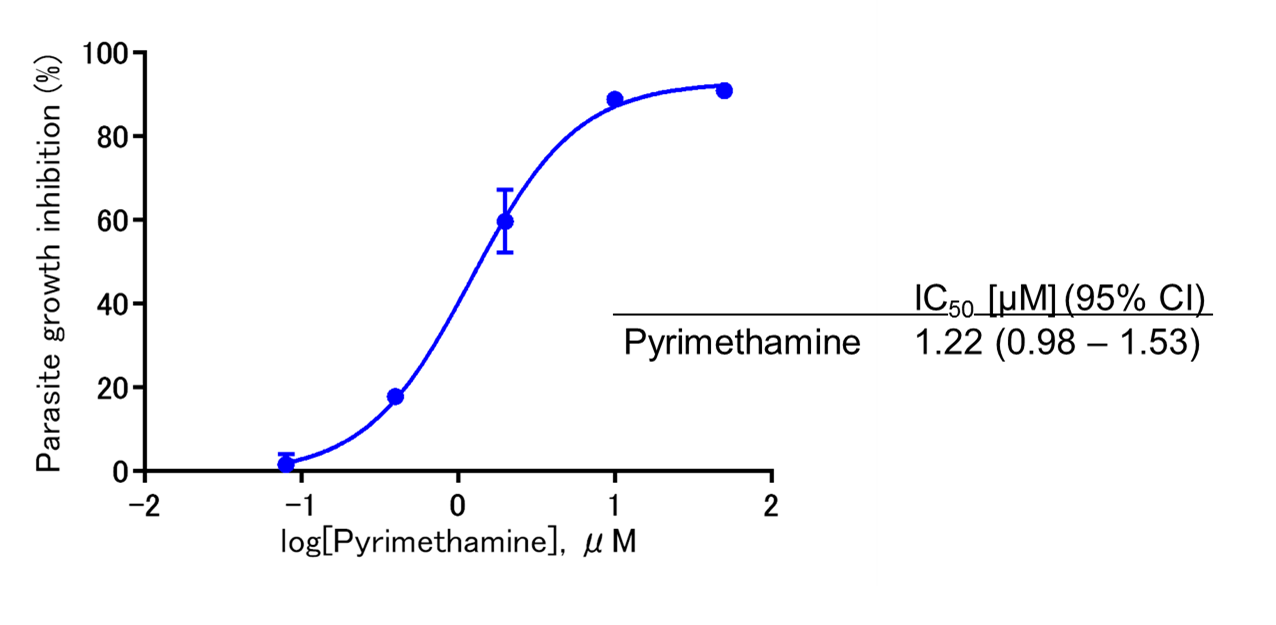

Supplement: S1 Fig — HFF cells infected with RH-2F parasites were incubated with various concentrations of pyrimethamine and parasite growth was measured by using the β-galactosidase assay. Inhibition rates were calculated using wells added DMSO (0% inhibition) and mock-infected wells (100% inhibition). (TIF) [file pone.0178203.s001.tif]

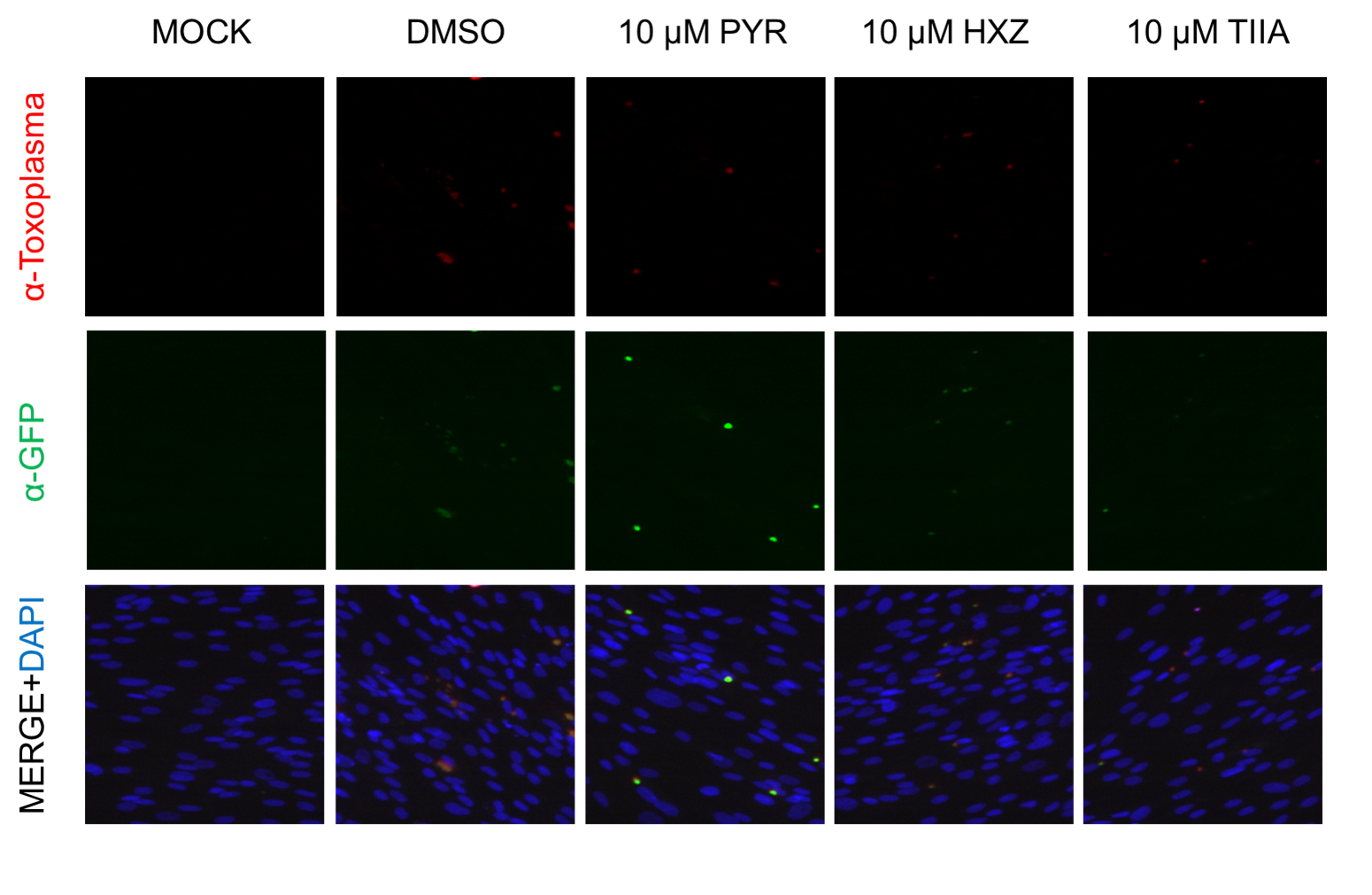

Supplement: S2 Fig — High resolution and individual channel of the images in Fig 3B are shown. PYR: Pyrimethamine, HXZ: Hydroxyzine, TIIA: TanshinoneIIA. (TIF) [file pone.0178203.s002.tif]

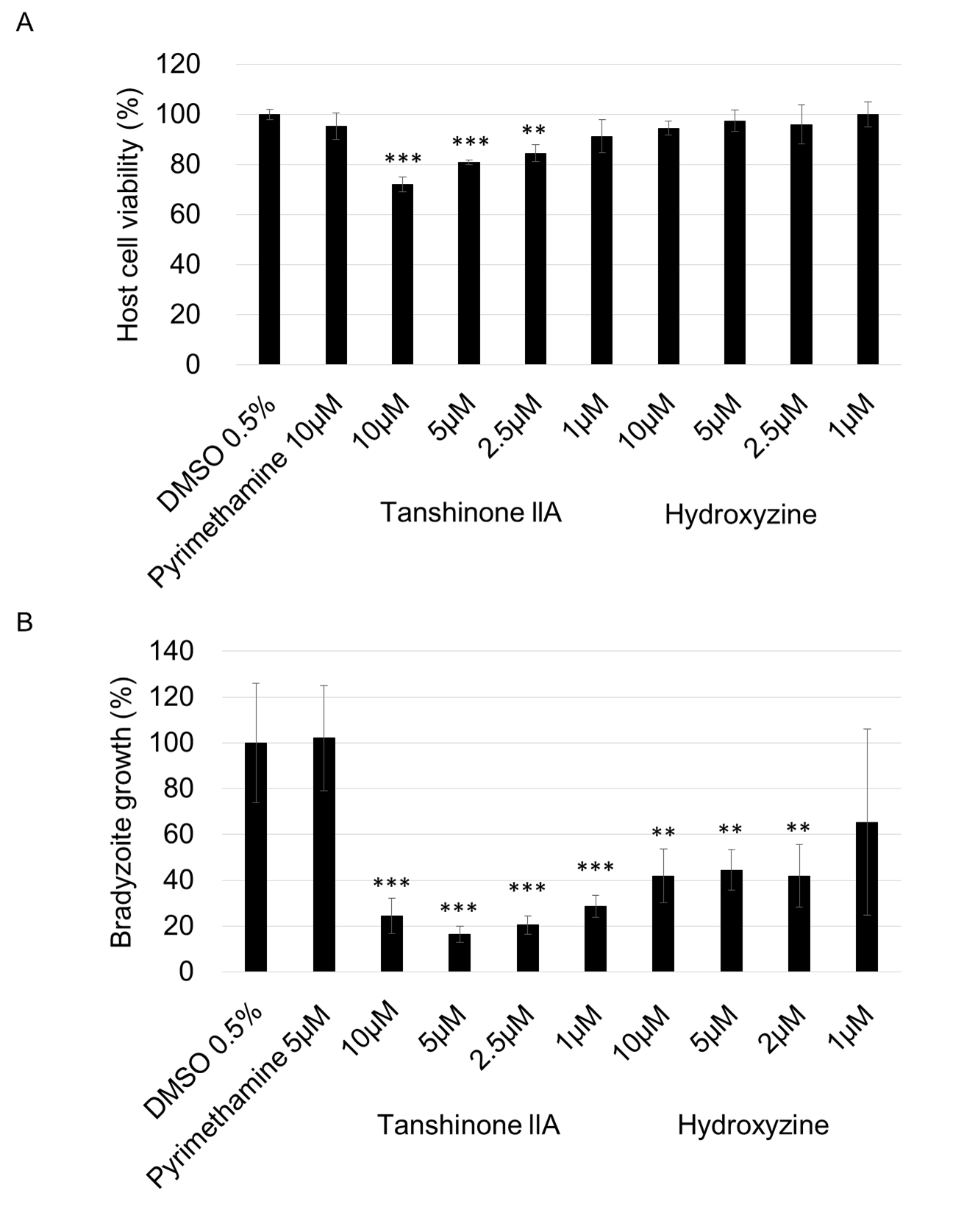

Supplement: S3 Fig — (A) HFF cells were incubated with bradyzoite condition (pH8.1, 0.03% CO2) with tested compounds. After 48 hours, host cell viabilities were measured. (B) PLK_DLUC_1C9 T. gondii were inoculated onto a monolayer of HFF cells and incubated for 2 h before undergoing bradyzoite induction for 3 days. After bradyzoite induction, compounds, as indicated, were added to the medium and infected host cells were incubated for 2 days under bradyzoite culture conditions. Firefly luciferase activities, under the control of the bradyzoite-specific BAG1 promoter, were measured and normalized to non-treated control (DMSO) wells. The statistical difference between the DMSO control and each compound was evaluated by using Dunnett’s test. ** p < 0.01 and *** p <0.001. (TIF) [file pone.0178203.s003.tif]
